# TMEM119 as a specific marker of microglia reaction in traumatic brain injury in postmortem examination

**DOI:** 10.1007/s00414-020-02384-z

**Published:** 2020-07-27

**Authors:** Simone Bohnert, Anja Seiffert, Stefanie Trella, Michael Bohnert, Luitpold Distel, Benjamin Ondruschka, Camelia-Maria Monoranu

**Affiliations:** 1 Institute of Forensic Medicine, University of Wuerzburg, Versbacher Str. 3, 97078 Wuerzburg, Germany; 2 Department of Radiation Oncology, Friedrich Alexander University of Erlangen-Nürnberg, Universitätsstr. 27, 91054 Erlangen, Germany; 3 Institute of Legal Medicine, University Medical Center Hamburg-Eppendorf, Butenfeld 34, 22529 Hamburg, Germany; 4 Department of Neuropathology, Institute of Pathology, University of Wuerzburg, Josef-Schneider Str. 2, 97080 Wuerzburg, Germany

**Keywords:** Cerebrospinal fluid, Forensic neuropathology, Forensic neurotraumatology, Immunohistochemistry, Immunocytochemistry, Biomarker

## Abstract

The aim of the present study was a refined analysis of neuroinflammation including TMEM119 as a useful microglia-specific marker in forensic assessments of traumatic causes of death, e.g., traumatic brain injury (TBI). Human brain tissue samples were obtained from autopsies and divided into cases with lethal TBI (*n* = 25) and subdivided into three groups according to their trauma survival time and compared with an age-, gender-, and postmortem interval-matched cohort of sudden cardiovascular fatalities as controls (*n* = 23). Brain tissue samples next to cortex contusions and surrounding white matter as well as samples of the ipsilateral uninjured brain stem and cerebellum were collected and stained immunohistochemically with antibodies against TMEM119, CD206, and CCR2. We could document the highest number of TMEM119-positive cells in acute TBI death with highly significant differences to the control numbers. CCR2-positive monocytes showed a significantly higher cell count in the cortex samples of TBI cases than in the controls with an increasing number of immunopositive cells over time. The number of CD206-positive M2 microglial cells increased survival time-dependent. After 3 days of survival, the cell number increased significantly in all four regions investigated compared with controls. In sum, we validate a specific and robustly expressed as well as fast reacting microglia marker, TMEM119, which distinguishes microglia from resident and infiltrating macrophages and thus offers a great potential for the estimation of the minimum survival time after TBI.

**Electronic supplementary material:**

The online version of this article (10.1007/s00414-020-02384-z) contains supplementary material, which is available to authorized users.

## Introduction

According to the Federal Statistical Office, more than 5000 individuals die from sequelae of traumatic brain injury (TBI) in Germany every year, and a relevant part of these deaths is forensically investigated under traumatological, reconstructive, and biomechanical aspects [1, 2]. For this purpose, a medicolegal routine commonly uses the methods of radiology, autopsy, and histology to determine whether there were brain injuries and whether they were causal for death [3]. Immunohistochemistry (IHC) has become an integral part of forensic histopathology over the last decades [4–6] and allows a more detailed approach in diagnosing and interpreting traumatic fatalities, when using biomarkers or proteins with high specificity to structures of the central nervous system (CNS) [7–11]. As additional tools in forensic neuropathological diagnosis, postmortem biochemical and immunocytochemical analyses of various cytokines, acute phase proteins, CNS biomarkers [8, 12–16], or Na^+^-glucose cotransporters [17] are increasingly performed in cerebrospinal fluid (CSF) and brain tissue and integrated into forensic assessments of these deaths.

In response to TBI, the brain orchestrates a complex immunological tissue reaction [18]. It has long been known that a diffuse microglial reaction follows after TBI, but this knowledge has rarely been linked to detailed timeframes and forensic questions before. Interestingly, the number of microglial cells is known to increase within a survival time of 24–72 h following TBI [19]. As the major cellular component of the innate immune system in the CNS and as the first cellular defense line whenever damage occurs (regardless if traumatic or not), microglia play a critical and strategic role in neuroinflammation [20–24]. Microglia are representatives of the mononuclear phagocyte series of cells as resident tissue macrophages such as in other organs (e.g., the Kupffer cells of the liver and the dendritic cells of the skin) [25]. In response to extensive tissue damage or any pathogens’ invasion, microglia can change into an amoeboid morphology, primarily acting in a phagocytic/macrophage fashion and being difficult to differentiate from infiltrating macrophages [26, 27].

One of the diagnostic challenges in the identification of microglia is that they are hardly noticeable in routine histology, and the absence of a specific reliable immunohistochemical marker to detect *all* microglia independent of their activation state aggravated this situation [25]. The expression pattern of the most widely used microglia marker (ionized calcium-binding adaptor molecule 1; Iba1 and CD68) in brain tissue slides does not allow any differentiation between resident microglia and infiltrating blood-derived macrophages [28]. Since 2016, TMEM119 is known as a microglia-specific and robustly expressed trans-membranous molecule, which is not expressed by other macrophages and immune or neuronal cells, and forms, therefore, the most promising microglia marker to date [28, 29]. TMEM119 has served as a reliable immunohistochemical microglia marker for neurodegenerative diseases since then [30] and was recently stained by an adapted protocol for immunocytochemistry even in postmortem CSF samples of trauma cases [30].

Differentiation of microglia activation is more complex, however. As in other CNS injuries, microglia activation in TBI results in different phenotypes [31]. On the one hand, the M1-like phenotype is integrated into the CNS inflammatory response by the expression of different surface markers, e.g., CD86, CD40, or inducible nitric oxide synthase (iNOS). On the other hand, the M2-like phenotype improves the phagocytic function and immunosuppression by the expression of the surface marker CD206 [32, 33].

The recruitment of bone marrow-derived monocytes into the injured brain parenchyma following TBI and their differentiation into macrophages complicate the interpretation of histological findings, especially bearing the ability of these cells to change its phenotype in mind [34]. Two subpopulations of bone marrow-derived monocytes have been defined by their cell surface expression of different chemokine receptors, namely, CCR2 and CX3CR1. While CCR2-positive cells are described as inflammatory monocytes, CX3CR1-labeled cells represent patrolling monocytes [35].

Given the difficulty to accurately evaluate microglial contributions to brain pathology in respect to its cellular diversity, the aim of the present study was to investigate TMEM119 for the first time as a useful microglia-specific marker in forensic assessments of traumatic causes of death, e.g., TBI, together with a detailed insight into the phagocytic function by CD206 immunostaining and the neuroinflammation capacity of monocytes using CCR2 immunostaining.

## Material and methods

### Sampling and processing

Human brain tissue samples were obtained from autopsies performed at the Institute of Forensic Medicine, University of Wuerzburg. This research study has been approved by the ethics committee of the Medical Faculty of the University of Wuerzburg (local number 203/15). Forty-eight cases were included in this study, 17 females and 31 males; the age at death ranged from 5 to 95 years. The samples were divided into cases with lethal TBI (total number *n* = 25: *n* = 4 primary brain damage and *n* = 21 secondary brain damage; case characteristics are displayed in detail in Table 1) and compared with a cohort of cardiovascular fatalities as controls (total number *n* = 23: *n* = 19 sudden cardiac death, *n* = 3 acute myocardial infarction, *n* = 1 ruptured aortic aneurysm) with their postmortem interval (PMI) varying between 1 and 13 days. The exact survival times of the TBI cases were known from the medical records or police investigations, which ranged between several minutes to 6 months; survival times of the control cases were defined as “none” given that no traumatic impact to the head had happened before death. The trauma cases were subdivided into three groups according to their trauma survival time being acute deaths after TBI (survival time < 2 h), subacute deaths after TBI (survival time > 2–72 h), and delayed deaths after TBI (survival time > 3 days). Exclusion criteria for sampling were as follows: the existence of former CNS injuries (“repetitive” trauma) or neurodegenerative diseases and putrefactive tissue changes.

**Table 1:** Characteristics of all traumatic brain injury (TBI) cases of this study

Case number	Sex	Age	TBI	Mechanism of death	Cortical contusion	Brain weight	Degree of edema
			< 2 h survival				
1	f	32	Immediately	Car accident	Frontal	1065 g	None
2	f	76	< 1 h	Fall	Frontal/temporal	1280 g	None
3	m	19	< 1 h	Car accident	Frontal	1220 g	None
4	m	19	Immediately	Car accident	Frontal	1370 g	None
			> 2–72 h survival				
1	m	50	< 12 h	Fall	Frontal/temporal	1500 g	Severe
2	m	75	4 h	Car accident	Frontal/temporal	1480 g	Severe
3	m	22	< 12 h	Fall	Temporal	1390 g	Moderate
4	m	77	4 h	Fall	Frontal/temporal	1590 g	Severe
5	m	59	5 h	Car accident	Frontal/temporal	1420 g	Moderate
6	f	80	24 h	Fall	Frontal/temporal	1150 g	None
7	m	42	2 h	Car accident	Frontal	1410 g	Moderate
8	m	87	24 h	Motor vehicle accident	Frontal	1460 g	Moderate
			> 3 days survival				
1	m	66	12 days	Car accident	Frontal/temporal	1560 g	Severe
2	f	20	5 days	Car accident	Frontal/temporal	1470 g	Severe
3	m	82	60 days	Fall	Frontal/temporal	1330 g	Moderate
4	m	67	9 days	Car accident	Frontal/temporal	1590 g	Severe
5	m	82	21 days	Fall	Frontal/temporal	1470 g	Moderate
6	m	88	17 days	Fall	Frontal/temporal	1090 g	None
7	f	76	4 days	Fall	Frontal/temporal	1120 g	None
8	f	80	4 days	Fall	Frontal/temporal	1190 g	None
9	m	88	24 days	Fall	Frontal/temporal	1180 g	None
10	f	87	42 days	Fall	Frontal/temporal	1220 g	None
11	f	95	4 days	Fall	Frontal/temporal	1430 g	Moderate
12	m	73	5 days	Car accident	Frontal	1500 g	Moderate
13	m	84	56 days	Motor vehicle accident	Frontal	1305 g	Moderate

Brain tissue samples next to macroscopically or microscopically verifiable cortex contusions as well as samples of the ipsilateral uninjured brain stem (pons) and ipsilateral uninjured cerebellum were collected and fixed in neutral buffered 10% formalin and then embedded in paraffin. The cerebral samples were counted in both, cortical layers and white matter. Brain stem and cerebellum samples were chosen as CNS regions being vulnerable to hypoxic episodes and processes [36–38]. After paraffinization, the wax blocks were sliced at 6 μm using a microtome. Consecutive sections were mounted on microscope slides and stained immunohistochemically as described previously [39] with commercially available antibodies against TMEM119 in a dilution of 1:1000 (Sigma, St. Louis, USA), against CD206 in a dilution of 1:500 (Bio-Rad, Marnes-la-Coquette, France) and CCR2 in a dilution of 1:200 (Abcam, Berlin, Germany). Control slides were stained by omitting the primary antibodies given above to test for unspecific staining in all staining charges. The microphotographs of the brain sections were taken with an Olympus DP 27 digital camera mounted on an Olympus BX508 microscope using × 100 magnification constantly (both Olympus Corporation, Tokyo, Japan).

For the slides with different antibodies investigated, five randomized images each per slide were taken to obtain a representative surface for all sections. The total surface of the images equals 10.75 mm^2^ (2.15 mm^2^ per single photograph). For the quantitative evaluation of the sections, an image processing software (Biomas, Erlangen, Germany) was used as described before [40]. Before the electronic count, parameters of cell morphology (size and staining intensity) were defined for each antibody, which were not changed throughout the evaluation. The software transferred the data automatically into an Excel macro table (Microsoft Corporation, Redmond, USA). The number of cells discernible in the five fields of view was set against the area investigated and calculated as the number of immuno-positive cells per square millimeter.

### Statistical analysis

The Excel version 16.15 (Microsoft Corporation) and GraphPad Prism software version 8 (GraphPad Software, La Jolla, USA) were used for statistical evaluation. The Shapiro-Wilk normality test was used to test the distribution of the samples. Parametric data of samples were then tested using an ordinary one-way ANOVA with post hoc Tukey’s multiple comparisons test subsequently. A Kruskal-Wallis test was used for nonparametric data followed by Dunn’s test to avoid repetitive testing failure. The Spearman coefficients were reported for the correlations, respectively. Adjusted *p* values equal to or smaller than 0.05 were considered statistically significant.

## Results

Descriptive analysis of typical forensic confounders such as age at death, sex of cadavers, brain weight at autopsy, and PMI until tissue sampling yielded no statistical differences between TBI cases and controls and matched each other (see Supplemental Table 1). There were moderate positive correlations between brain weight and sex of the traumatized individuals (*r* = 0.47) and between brain weight and PMI in TBI cases (*r* = 0.59) (see Supplemental Table 2**)**.

**Fig. 1:**
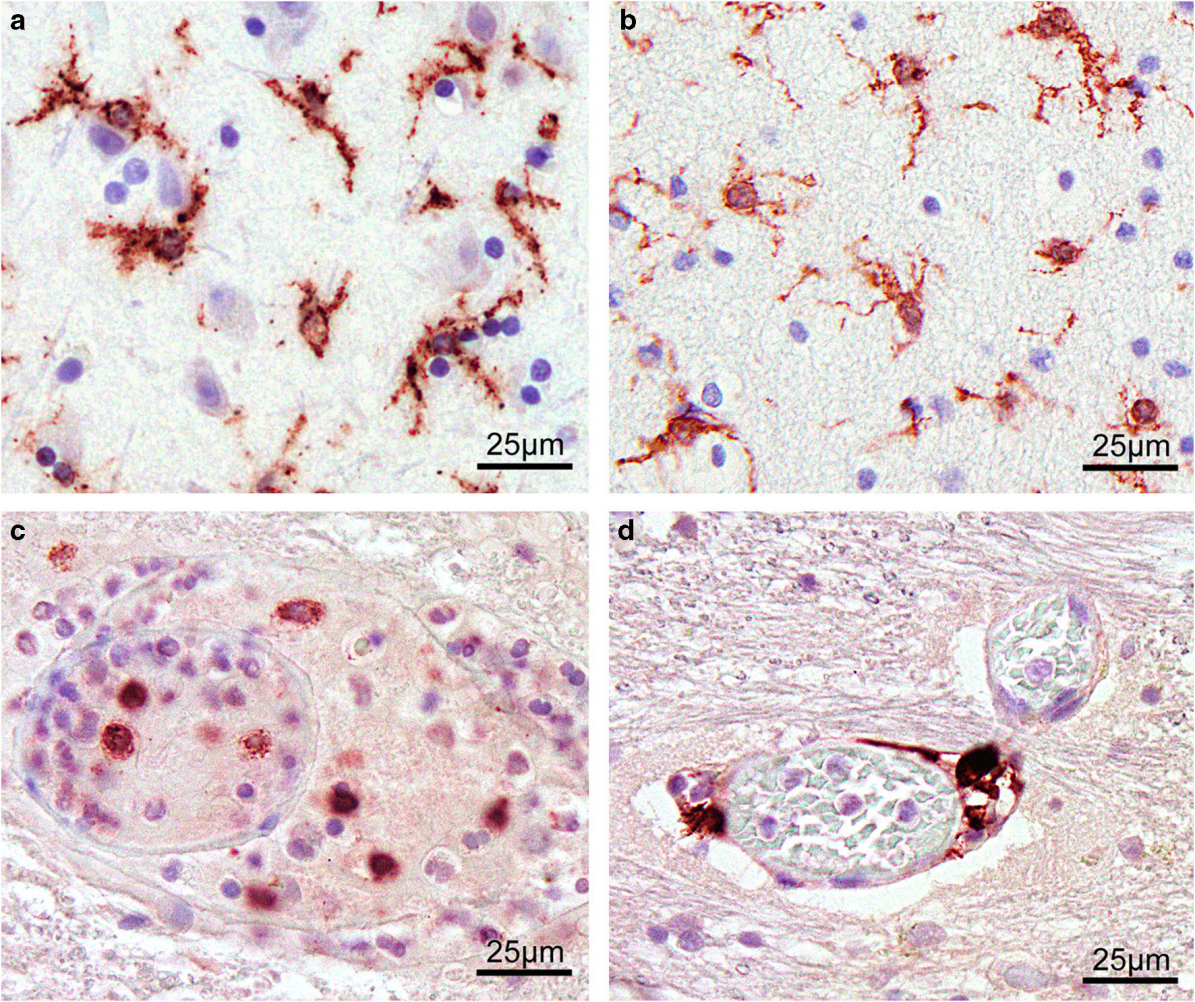
Examples of immunohistochemical staining results using TMEM119 in the cortex (A) and the white matter (B) in a traumatic brain injury (TBI) case with a survival time of less than 2 h. CCR2 decorates microglia in the pons in a subacute death case (C) and CD206 is immunopositively stained in the cerebellar perivascular space as illustrated in a TBI fatality with survival of more than 3 days (D). Magnification: × 400

Typical IHC staining results of all three markers used and different regions investigated are given in Fig. 1**.** To illustrate cell-specific staining patterns per immunohistochemical marker in detail, the here presented staining examples are magnified × 400.

**Fig. 2:**
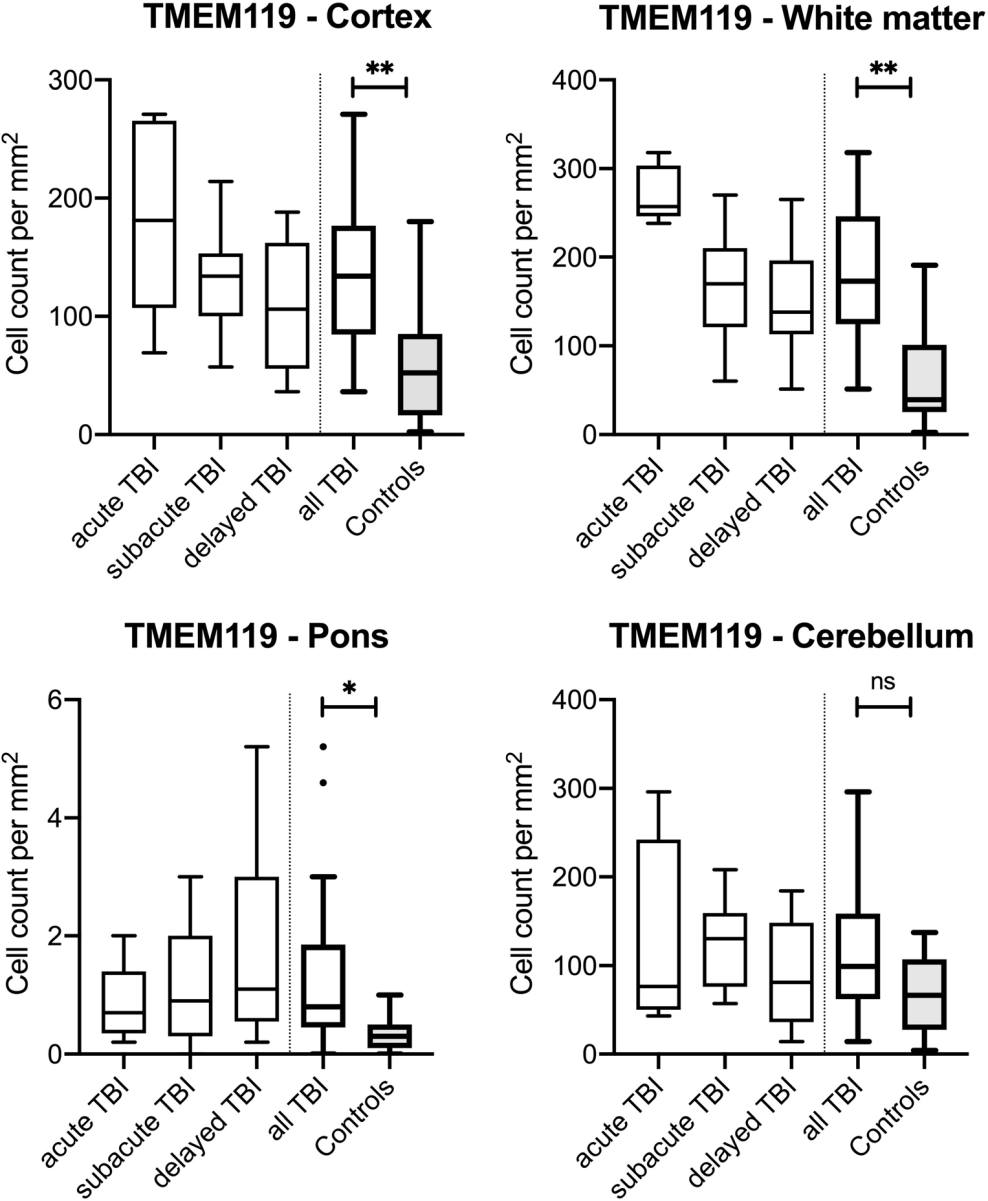
Box plot diagrams displaying the different total numbers of TMEM119-positive microglial cells (counted in five digital images at a × 100 magnification) depending on the survival time of traumatic brain injury (TBI) fatalities compared with the controls in the four brain regions investigated. The solid black lines indicate the median, and the outlines of the boxes the 25th and 75th percentile. Whiskers are defined as Tukey’s end of 1.5 times interquartile range and all outliers are illustrated as dots outside these fences. *p < 0.05; **p < 0.001 (illustrated for “all TBI” vs. controls only); ns, not significant

In more detail, TMEM119-positive microglia was stainable in all cases and regions, irrespective of the cause of death. The lowest cell counts were achieved for control cases in all four regions. Without showing significant differences in the cerebellum, the TBI cell counts were highly statistically elevated compared with controls in the cortex (*p* < 0.0001), the white matter (*p* < 0.0001), and the pons (*p* = 0.0003) (see Fig. 2).

When looking at the different survival time categories, these three regions showed comparable changes over time. The highest numbers of TMEM119-positive cells were counted in the acute death cases (all with highly significant differences to the control numbers). Even the cell numbers in the subacute TBI cases stayed significantly higher than in controls in the cortex (*p* = 0.0028), the white matter (*p* = 0.0077), and the pons (*p* = 0.0029). When reaching the delayed death time interval, the cell counts decrease to the level of control cases showing no longer any significant differences (*p* ≥ 0.0536).

**Fig. 3:**
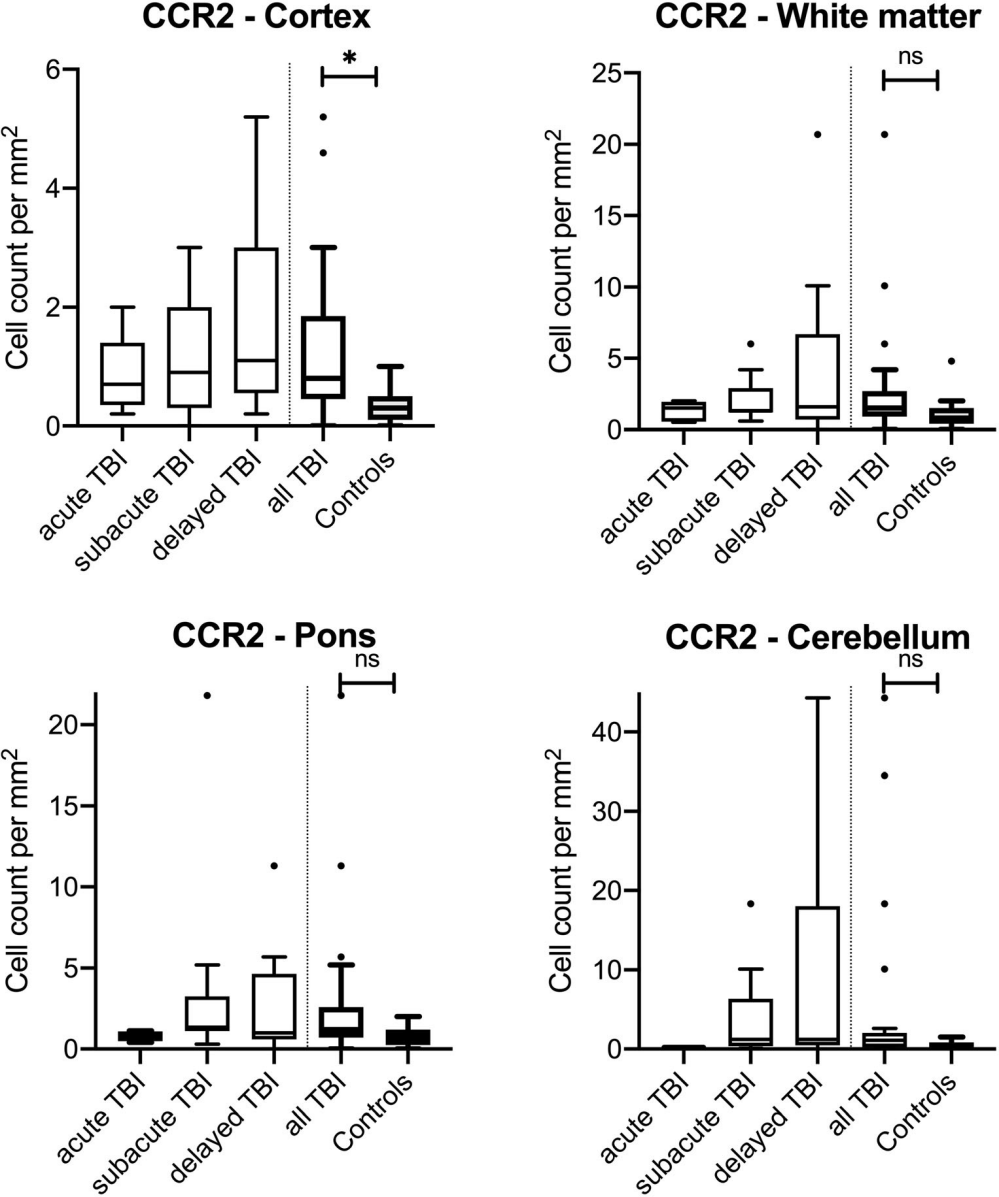
Box plot diagrams displaying the different total numbers of CCR2-positive monocytes (counted in five digital images at a × 100 magnification) depending on the survival time of traumatic brain injury (TBI) fatalities compared with the controls in the four brain regions investigated. The solid black lines indicate the median, and the outlines of the boxes the 25th and 75th percentile. Whiskers are defined as Tukey’s end of 1.5 times interquartile range and all outliers are illustrated as dots outside these fences. *p < 0.05 (illustrated for “all TBI” vs. controls only); ns, not significant

CCR2-positive monocytes showed sparse immunostaining throughout the regions investigated. Some slides were completely negative for CCR2 in both, TBI and control samples. In the cortex samples, cell counts in TBI cases were significantly higher than in controls (*p* = 0.0015) with increasing immunopositive cell numbers over time. While the number in acute death stayed non-significantly different to the cardiovascular cases (*p* = 0.7862), the other survival time categories showed significantly higher cell numbers (subacute deaths: *p* = 0.0449; delayed deaths: *p* = 0.0108). Counting in white matter, pons and cerebellar samples showed no relevant differences between the causes of death for CCR2 staining (see Fig. 3).

**Fig. 4:**
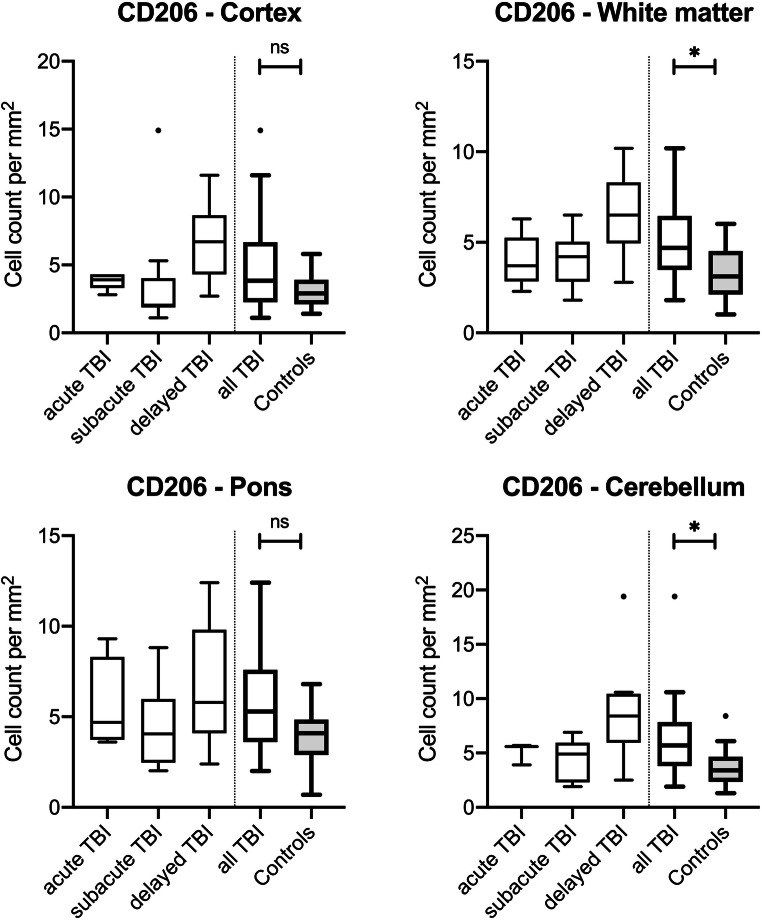
Box plot diagrams displaying the different total numbers of CD206-positive M2 microglial cells (counted in five digital images at a × 100 magnification) depending on the survival time of traumatic brain injury (TBI) fatalities compared with the controls in the four brain regions investigated. The solid black lines indicate the median, and the outlines of the boxes the 25th and 75th percentile. Whiskers are defined as Tukey’s end of 1.5 times interquartile range and all outliers are illustrated as dots outside these fences. *p < 0.05 (illustrated for “all TBI” vs. controls only); ns, not significant

CD206-positive M2 microglial cells were distributed in comparable with low numbers throughout control tissue (medians below 5 cells/mm^2^), irrespective of the brain area. After TBI, the number of CD206-positive cells changed with increasing survival time: A slight initial increase in cell numbers of acute death cases (non-significant in all four regions investigated) is followed by comparable cell numbers in subacute death cases without significant differences in cell numbers between acute and subacute death cases (all *p* > 0.90). After 3 days of survival, the cell number increases significantly in cortex samples (*p* = 0.0202), the white matter (*p* = 0.0002), the pons (*p* = 0.0332), and the cerebellum (*p* = 0.0009) compared with the control counts. When comparing all TBI cases with the controls by CD206, significant differences were calculated for the regions of white matter (*p* = 0.0205) and the cerebellum (*p* = 0.0454); for the box plot presentation, see Fig. 4.

By correlative comparisons of the staining results in all four regions, only the negative correlation between TMEM119- and CCR2-positive cells in the pons of TBI cases was proven to be significant (*r* = − 0.452; *p* = 0.031). Staining results in control cases showed no confounding influence of sex, age, brain weight, and PMI for any of the three markers TMEM119, CCR2, and CD206 (see Supplemental Table 3). However, age at death negatively correlated to TMEM119 immunostaining in white matter trauma samples and the brain weight showed significantly positive correlations to CCR2 immunopositivity in the cerebellum and pons, but a negative correlation to CD206 staining in the cerebellum (see Supplemental Table 2).

## Discussion

The present study is a refined analysis of microglia response in postmortem brain tissue after TBI with different survival times. In this investigation, we could use the microglia-specific antibody TMEM119 to document the direct and immediate neuroinflammatory response of brain tissue after TBI compared with cardiovascular controls and to further analyze to what extent infiltrating myeloid cells were involved, using the antibody CCR2. Moreover, we were able to obtain information on the role and time dependency of M2-polarized CD206-positive microglia in response to TBI.

In the region of the cerebral cortex and the surrounding white matter, significant differences were found for TMEM119-positive cells, even in TBI cases with survival times less than 2 h. In contrast, hypoxia-sensible areas farther away from the directly impacted cortical area (pericontusional zone) such as the brain stem showed a weaker manifestation of TMEM119-positive cells compared with the controls until the survival time reached 3 days. The cerebellum, which was the farthest away from the site of trauma in the given study and additionally protected anatomically by its falx, did not show any evidence of increased microglial cell numbers after trauma. Thus, the marker TMEM119, which was used for the very first time in the forensic assessment of TBI to the best of the author’s knowledge, allows conclusions both as to the age of the injury (highest cell count already after a survival time of less than 2 h) and, in contusions not macroscopically discernible, on the precise localization of traumatized tissue (highest cell count in the pericontusional zone).

To document the complexity of the immune response in the traumatized brain tissue after mechanical damage, the antibody CCR2 was further used to detect inflammatory bone marrow-derived monocytes [41]. It has been reported that monocytes circulating between the two compartments blood and bone marrow are recruited into the traumatized cerebral parenchyma by the breakdown of the blood-brain barrier (BBB) as the most commonly used hypothesis. However, a newer study has shown that these pooled monocytes typically “enter” the CNS via the soft meninges of the spinal cord, which are directly adjacent to the site of trauma [42]. These CCR2-positive monocytes are predominantly localized in the injured region within 3 days after the trauma [34]. The data presented here now demonstrate that in the cerebral cortex, the number of CCR2-positive cells increases significantly with a more prolonged survival time. This observation may support the assumption that CCR2-positive cells fire up the inflammatory cascade and give an impulse for a perivascular migration of leukocytes (leukodiapedesis) and further immune cells into the damaged brain parenchyma. CCR2-positive cells only showed relevant differences in the traumatized tissue, illustrating the staining as being a possible marker of direct impact. We could demonstrate a negative correlation between CCR2 and TMEM119 in the pons region and higher CCR2 cell numbers with higher brain weight, which does not allow a valid conclusion given the lack of further relevant correlations. Thus it remains unclear whether immune cells generally infiltrate the brain tissue from the periphery (*general* reaction) after local or global irritation or whether there is a *specific* “cutoff” level, e.g., for the local immune response of microglia, which is responsible for the scope of infiltration of immune cells from the periphery (*specific* reaction depending on this threshold).

As in other CNS diseases, it seems that according to a simplified concept, microglia activation following TBI results in two phenotypes, which either have an inflammatory effect (M1) or a regenerative effect (M2) depending on their imprinting and environment. The pro-inflammatory M1 promotes the production of cytokines, which induce neuroinflammation in the traumatized tissue [43] resulting in an acute phase response with various biochemical marker changes. In contrast, there are microglia/macrophages of the M2 type which show an immunosuppressive effect and thus contribute to the regeneration of the damaged tissue by their phagocytic activity [18]. This point of view in polarization states of microglia has changed the pharmacological treatment concept of TBI only a few years ago. Strategies inhibiting the M1 phenotype (neuroinflammation) and promoting the M2 phenotype (neuroregeneration) of microglial cells could alleviate cerebral cell damage in a broader variety of brain injury animal models. The following pharmacological studies must not only try to regulate the ratio of M1 and M2 microglia and therapies by focusing only on suppressing microglia/macrophage activation but also support regenerative pathways [44].

In the last few years, the postulate of two existing microglia phenotypes and simplificated “counteracts” was sustainably attacked by extensive RNA transcription analyses [45, 46]. In an animal TBI model, evidence was provided for mixed states of microglia described as *Mtrans* [47]. However, this was not translated in human microglia research to date. Moreover, the role of infiltrating macrophages and other immune cell polarization seems relevant in the context of microglia dynamics after TBI and their dependence on different brain injury types, such as (i) focal or diffuse or (ii) acceleration or deceleration. To date, the majority of the existing data on M1-/M2-like phenotypes after TBI have been developed using *focal* TBI mouse models showing significant tissue damage/loss and robust cellular infiltration but human data is lacking. *Diffuse* brain injury animal models, such as the midline fluid percussion injury (mFPI) model, also cause chronic microglial activation with M1-like characteristics but without relevant contributions from infiltrating macrophage populations [48]. In this study, we were able to document a direct and immediate response of resident microglia, a delayed migration of the initially perivascular localized M2 phenotype and the recruitment of bone marrow-derived monocytes into the injured brain parenchyma following TBI. These study observations could be explained best as complex and multicellular immunological reaction in traumatized human brain tissue, irrespective of how this trauma occurred, or which pathways of secondary brain damage are activated. The expression patterns in realistic human head traumas are only partly comparable with the results of laboratory animal models.

We decided to use the antibody CD206 in this study to immunostain predominantly perivascular, “healthy” M2 microglia with enhanced phagocytic activity and reduced production of inflammatory mediators [49, 50]. The main reason for this decision was the preference of CD206 for immunosuppressive M2 microglia known from studies on glioblastomas. Here, the proliferation of glioma cells and tumor neoangiogenesis due to hypoxia are mediated by a prevalence of M2 microglia [51]. The study focus was set on M2 microglia, which have been less investigated so far in this context, and we could show that the number of CD206-positive cells increases in the investigated brain areas compared with the control group after a longer survival time. This supports the assumption that there is a delayed migration of the initially perivascular localized M2 cells into the damaged tissue. Moreover, the present results support clinical observations of M2 phenotype microglia equipped with higher potential in clearing cell debris and, therefore, may represent an endogenous effort to clean injured brain tissue and restrict brain damage locally by an accumulation of oligodendrocyte progenitor cells, a reactive astrogliosis resulting in a pronounced white matter recovery after TBI [47, 52, 53]. The significant rise of CD206-positive cells in the cerebellum and the white matter following TBI may be due to hypoxia as part of the secondary brain damage, knowing that this pathological condition stimulates cell proliferation of M2 microglia in the brain tissue.

## Limitations

In our investigation, we used heterogeneous study material with a wide age range and differing PMI, which may influence the distribution pattern of the examined cells in the brain but represents the realistic tissue quality of our daily autopsy material. Moreover, different formaldehyde and paraffin fixation times of the tissues may have led to different color reactions and thus to different expression patterns of the cells examined. At the beginning of this given study, a control group of cardiovascular fatalities was determined, whose preselection did not allow a comparison of the results presented to other causes of death, especially in the cases of hypoxic brain damage such as strangulation but enabled us to keep the study material within reasonable limits.

## Conclusion

Summarizing, this study validated a specific and robustly expressed as well as fast reacting microglia marker, TMEM119, which distinguishes microglia from resident and infiltrating macrophages for its beneficial use in TBI brain tissue investigation. CCR2 and CD206 are useful indicators of microglial invasion in the cases of longer TBI survival times. Thus, the investigation of microglia markers offers great potential in the estimation of the minimum survival time after TBI and to demonstrate trauma-reactive mechanisms in the CNS.

## Electronic supplementary material

ESM 1 (DOCX 15 kb)
