# Supplementary material for: TMEM119 as a specific marker of microglia reaction in traumatic brain injury in postmortem examination
Source: Int J Legal Med. 2020 Jul 27;134(6):2167–76. doi: 10.1007/s00414-020-02384-z (PMC7578160; doi:10.1007/s00414-020-02384-z)
Supplement: Supplementary file 1 — (DOCX 15 kb) [file 414_2020_2384_MOESM1_ESM.docx]

| Analysis | P value | Test used |
| --- | --- | --- |
| Age TBI vs. controls | p = 0.0523 | Mann-Whitney U test |
| Sex TBI vs. controls | p = 0.6058 | Chi-square test |
| Brain weight TBI vs. controls | p = 0.8454 | Mann-Whitney U test |
| PMI TBI vs. controls | p = 0.7089 | Mann-Whitney U test |

**Supplemental Table 1:** Descriptive analysis of traumatic brain injury (TBI) cases versus (vs.) controls. PMI, post-mortem interval.

|  | Sex | Age | Brain weight | PMI |
| --- | --- | --- | --- | --- |
| Sex TBI | 1,00 | -0,13 | **0,47** | 0,23 |
| Age TBI | -0,13 | 1,00 | -0,19 | -0,31 |
| Brain weight TBI | **0,47** | -0,19 | 1,00 | **0,59** |
| PMI TBI | 0,23 | -0,31 | **0,59** | 1,00 |
| TMEM119 Cortex | 0,02 | -0,08 | 0,21 | 0,30 |
| TMEM119 WM | -0,05 | **-0,51** | 0,20 | 0,26 |
| TMEM119 Cerebellum | -0,02 | -0,21 | 0,02 | 0,38 |
| TMEM119 Pons | 0,06 | 0,06 | 0,07 | 0,14 |
| CCR2 Cortex | 0,21 | -0,25 | 0,01 | -0,03 |
| CCR2 WM | -0,08 | -0,16 | 0,37 | 0,29 |
| CCR2 Cerebellum | 0,38 | 0,18 | **0,54** | 0,23 |
| CCR2 Pons | 0,23 | -0,07 | **0,44** | 0,40 |
| CD206 Cortex | -0,01 | 0,15 | -0,28 | -0,15 |
| CD206 WM | -0,13 | 0,41 | -0,18 | -0,06 |
| CD206 Cerebellum | -0,21 | 0,25 | **-0,46** | -0,30 |
| CD206 Pons | -0,02 | 0,24 | -0,04 | -0,02 |

**Supplemental Table 2:** Spearman’s correlation matrix between potential confounders and counting results in TBI cases (correlation coefficient printed bold are statistically significant). PMI, post-mortem interval; WM, white matter.

|  | Sex | Age | Brain weight | PMI |
| --- | --- | --- | --- | --- |
| Sex Control | 1,00 | 0,32 | 0,14 | 0,27 |
| Age Control | 0,32 | 1,00 | 0,00 | 0,08 |
| Brain weight Control | 0,14 | 0,00 | 1,00 | -0,05 |
| PMI Control | 0,27 | 0,08 | -0,05 | 1,00 |
| TMEM119 Cortex | 0,13 | -0,27 | -0,09 | -0,11 |
| TMEM119 WM | 0,15 | 0,13 | -0,36 | 0,13 |
| TMEM119 Cerebellum | 0,01 | 0,17 | -0,38 | 0,08 |
| TMEM119 Pons | 0,02 | 0,08 | -0,16 | 0,16 |
| CCR2 Cortex | -0,03 | 0,02 | -0,30 | -0,31 |
| CCR2 WM | -0,18 | 0,15 | 0,08 | -0,26 |
| CCR2 Cerebellum | -0,01 | 0,10 | -0,09 | -0,22 |
| CCR2 Pons | 0,17 | 0,29 | -0,13 | 0,08 |
| CD206 Cortex | -0,34 | -0,16 | 0,14 | -0,22 |
| CD206 WM | -0,13 | 0,16 | -0,18 | 0,01 |
| CD206 Cerebellum | 0,30 | 0,07 | 0,05 | -0,14 |
| CD206 Pons | 0,31 | 0,35 | 0,39 | -0,27 |

**Supplemental Table 3:** Spearman’s correlation matrix between potential confounders and counting results in control cases (no correlation coefficient was statistically significant). PMI, post-mortem interval; WM, white matter.
